# Priorities for rapid and cost-effective vaccines to improve outbreak responses

**DOI:** 10.1371/journal.pbio.3003114

**Published:** 2025-04-07

**Authors:** Sarah C. Gilbert

**Affiliations:** 1 Nuffield Department of Medicine, Pandemic Sciences Institute, University of Oxford, Oxford, United Kingdom; 2 Chinese Academy of Medical Science (CAMS), Oxford Institute (COI), University of Oxford, Oxford, United Kingdom

## Abstract

The use of vaccine platform technologies greatly reduces the time and money required to develop a novel vaccine against an infectious disease. However, there is much still to do; this Perspective outlines vaccine development priorities to improve our response to future outbreaks.

## Teaser

The use of vaccine platform technologies greatly reduces the time and money required to develop a novel vaccine against an infectious disease, but much work is still needed if vaccines are to protect the world from the next pandemic.

Prior to 2020, when vaccines were typically developed from the pathogen itself via attenuation or inactivation, the path to vaccine licensure and rollout took a minimum of 4 years (but frequently much longer). Employing vaccine platform technologies enables much of the development work to be conducted prior to the creation of a vaccine against a specific pathogen, thus greatly reducing the time and expense required to reach vaccine approval. Although the licensure of multiple vaccines against SARS-CoV-2 in under 1 year was impressive, it became apparent that there is much work to be done to capitalize on the gains made if the world is to be better protected next time a pandemic begins.

A vaccine platform technology enables a “plug and play” approach to developing a new vaccine. One example of a platform technology is replication-deficient adenoviral vectors such as Ad26 or ChAdOx1. To produce a new vaccine, the genetic coding sequence of the novel vaccine antigen, taken from the infectious pathogen, is inserted into the adenoviral genome. Vaccine production then follows a standard approach that has already been optimized, along with other aspects such as storage temperature, shelf-life and the dose to be administered. A second example of a vaccine platform ia mRNA-based vaccines, consisting of messenger RNA encoding the vaccine antigen delivered inside a lipid nanoparticle. Together, the mRNA vaccines and adenoviral-vectored vaccines saved an estimated 16 million lives in the first year of use during the COVID-19 pandemic [1]. The advantages of platform technologies for rapid response are clear, because much of the work is done to optimize the platform in advance of using it to make a new vaccine; however, there is much more that needs to be achieved in order to respond rapidly and effectively when new disease outbreaks occur (Box 1).

**Box 1. Considerations for effective outbreak responses**

- We need international antibody standards for each disease
- We need to better understand the immune protection needed for each disease
- We need to be able to convert vaccine immunogenicity to efficacy for each disease
- Mucosal immunity should be studied
- If we don’t manufacture, we can’t vaccinate
- Thermostability matters. Vaccine supply matters. Cost per dose matters.
- Many factors contribute to saving many lives
- There is no single approach that will work for every disease

The World Health Organization list of Priority Pathogens, developed after the 2014 Ebola outbreak in West Africa, has now been replaced with a far more comprehensive document describing Priority Pathogen Families (“*Pathogens prioritization: a scientific framework for epidemic and pandemic research preparedness*”). Grouping the many thousands of pathogens with potential to infect humans into families not only allows us to take an exemplar from each family and develop the knowledge and resources to be able to respond to an outbreak with that pathogen, but also to understand what would be needed if the outbreak was caused by a family member of that pathogen. In 2020, our ability to protect against SARS-CoV-2 infection was hampered by the fact that, although four seasonal coronaviruses regularly cause infections in humans, there was no vaccine against any of them. Therefore, we had no prior knowledge of what a vaccine against a novel coronavirus would need to achieve to prevent transmission, infection, mild or severe disease, or death. Although vaccine development got off to a rapid start with preliminary immunogenicity data available by July [2,3], it was necessary to expand the trials to vaccinate thousands of participants and determine efficacy of the vaccine against mild disease, which took several more months before the first efficacy results were announced [4].

Already in 2025, there have been outbreaks of Marburg and Sudan filoviruses, Middle East Respiratory Syndrome (a coronavirus), Zika (a flavivirus) and Chapare hemorrhagic fever (an arenavirus). Looking ahead to the next time we have a large outbreak threatening to become a pandemic, there is much that we should be doing in preparation.

We need a better understanding of immune protection for each pathogen family. Ideally, correlates of protection will have been determined for at least one pathogen per family. A correlate of protection is the output of an immunoassay that predicts whether a person who has been vaccinated will be protected against a particular disease [5]. Correlates may be determined from a vaccine efficacy trial, comparing immune responses in those who were or were not protected from disease, or in preclinical studies. For example, a serum antibody titer of greater than 0.5 International Units (IU)/ml is accepted as providing protection against rabies [6].

To facilitate the definition of correlates of protection against further pathogens, we need international antibody standards and validated immunoassays. An international standard is a reference material derived from a pool of seropositive samples and assigned a nominal potency value. Comparing the output of immunoassays to the result produced by the international standard allows a numerical value in IU/ml to be assigned to each sample being tested. However, the immunoassays must produce reliable data, and before being employed to test serum samples must have undergone validation to determine features such as robustness, precision and limits of detection [7]. Validation of immunoassays and generation and testing of international standards both take time, but once established, allow direct comparison of immunology data generated in different clinical trials, which was not possible in 2020. Understanding the correlates of protection would allow early immunogenicity data from clinical trials to be used to predict vaccine efficacy, with rapid emergency use licensure, vaccine rollout, and post-roll-out surveillance then replacing phase III clinical trials.

However, accelerating the decision to roll-out vaccines will be of little practical use if there is not a sufficient supply of the vaccine, at an affordable price. Early vaccine deployment in the outbreak area could in theory prevent an outbreak from becoming a pandemic, thereby obviating the need to vaccinate the entire world and substantially reducing the number of vaccine doses required. But along with the many other challenges in achieving that aim, there would need to be sufficient supply of vaccine for early deployment. For known pathogens, a vaccine stockpile could have been prepared in advance, correlates of protection determined in nonhuman primate studies, and a phase II trial completed to determine if the required antibody titer can be achieved by vaccination of humans of different ages. The stockpile could then be deployed within a preagreed clinical trial protocol to test vaccine efficacy during the outbreak. Development of such protocols, including consideration of the use of placebo or delayed vaccination in order to determine vaccine efficacy should happen in ‘peace time’. However, even in that ideal scenario, vaccine manufacturing should be rapidly reinstated in case the stockpile is not sufficient to contain the outbreak. In the case of a novel pathogen, manufacture of a novel vaccine using the same design as one known to be effective against a closely related pathogen would be initiated, requiring a manufacturing facility on standby for such an eventuality. In parallel, tests on stored serum from earlier clinical trials with the stockpiled vaccine could be conducted to assess whether cross-reactive immune responses may provide any useful vaccine efficacy until a specific vaccine against the new pathogen becomes available. Hyper-local and mobile vaccine manufacturing units could play a part, with a facility on the back of a truck producing vaccines in the location they are needed; although quality control to meet regulatory standards must be considered.

Along with vaccine supply, delivery is needed before vaccination can be achieved.

Improvements in thermostability, allowing vaccines to be stored and transported without refrigeration, and the ability to administer vaccines without requiring a needle and syringe, would greatly facilitate vaccine rollout. Clinical trials to test mucosal vaccine delivery and determine appropriate correlates of protection are needed, as are more trials to understand the optimal vaccination regimen. The question of vaccine safety in pregnancy, or in people with immune systems compromised by HIV infection, cancer or age should be addressed for each of the vaccine platforms. This would reduce the changing messages, particularly regarding vaccination during pregnancy, that hampered COVID-19 vaccine uptake. Although SARS-CoV-2 infection during pregnancy is associated with an increased risk of severe illness and adverse perinatal outcomes, it took time to establish vaccine safety and efficacy, with recommendations to vaccinate or not changing over time, and a lower vaccine uptake than in the general population [8]. More generally, vaccine confidence must be restored, with people in all countries recognizing the value of vaccination.

We should not only focus on a fast response once an outbreak happens. We must keep up the momentum in developing and licensing vaccines for emergency use in advance of the next outbreak. Ebolavirus had caused multiple small outbreaks since the virus was first identified in 1976, before the West African outbreak in 2013–2016 claimed the lives of more than 11,000 people. If we are not well prepared enough to act quickly, pandemic response becomes very complicated as the pathogen evolves [9], and very expensive. No one anywhere in the world will be safe until everyone is safe. As any vaccinologist knows, an ounce of prevention is worth a pound of cure.
